# Basis-driven learnable operator for MLP-mixers

**DOI:** 10.3389/frai.2026.1769436

**Published:** 2026-08-24

**Authors:** Ahmed Elsheikh, Mohammed E. Fouda, Ahmed M. Eltawil

**Affiliations:** 1 Mathematics and Engineering Physics Department, Faculty of Engineering, Cairo University, Giza, Egypt; 2 Compumacy for Artificial Intelligence Solutions, Giza, Egypt; 3 CEMSE Division, King Abdullah University of Science and Technology, Thuwal, Saudi Arabia

**Keywords:** deep learning, learnable operators, learnable spectral transformation bases, MLP-mixers, neural networks

## Abstract

**Introduction:**

Existing multi-layer perceptron (MLP)-mixer architectures either rely heavily on extensive training data or employ rigid, hand-engineered mixing operations. This study introduces a Basis-Driven Learnable Operator (BDLO) for MLP-mixers, targeting a balance between high-capacity flexible learning and computationally efficient, yet constrained, handcrafted solutions.

**Methods:**

BDLO is a drop-in replacement for the shifting block of shifting-based MLP-mixers. It approximates shifting-based mixing operations by learning the coefficients of a real, complete, discrete basis, yielding an input-dependent transformation matrix that is applied to both rows and columns of the token table, while the patch embedding, channel-mixing MLPs, skip connections and classification head are left unchanged. The operator was integrated into CycleMLP, HireMLP and AS-MLP at three model sizes each, and all models were trained from scratch under an identical configuration on CIFAR10, CIFAR100, a reduced ImageNet (32 × 32, 500 classes) and full ImageNet1K. Standard and discrete cosine transform bases were compared, hyperparameter sensitivity was assessed with Optuna, and differences were tested using the Wilcoxon signed-rank test.

**Results:**

BDLO reduced the cost of the mixing layer to 1.68 GFLOPs, against 3.33–5.08 GFLOPs for the original layers, and reduced whole-model parameter counts by 21.8%–56.7% (mean reduction 12.84M), with a mean accuracy difference of 0.38% in favor of BDLO. The Wilcoxon test confirmed a significantly lower parameter distribution (*p* = 0.0039) and no statistically significant accuracy difference (*p* = 0.496 on CIFAR100, *p* = 0.0625 on CIFAR10, *p* = 0.5 on the reduced ImageNet). Results were invariant to the choice of basis (mean cosine distance 0.0049 between the learned coefficient vectors) and insensitive to the BDLO-specific hyperparameters.

**Discussion:**

Comparability holds in aggregate and in the parameter-constrained regime, with model-specific exceptions for baselines that include channel mixing (HireMLP) and for high-capacity baselines on larger datasets (AS-MLP). BDLO behaves as an input-adaptive spectral modulator whose bounded coefficients provide implicit regularization. This confirms the effectiveness of BDLO as an efficient operator replacement for shifting-based MLP-mixers, most suitable for parameter-constrained, small-to-medium-scale models in image recognition tasks.

## 1 Introduction

Several Neural Network (NN) architectures have gained popularity in specific research areas at different times. In computer vision, Convolutional Neural Networks (CNNs) have been the primary vision models (Li et al., 2021). Their main merit is the ability to combine features locally within a fixed-shaped sliding window. Recently, motivated by advancements in the field of Natural Language Processing (NLP), Transformers were integrated into the computer vision domain (Han et al., 2022). Transformers, constructed using self-attention layers that compute pairwise attention, are typically represented by the affinities of features in the embedding space. Recent validations indicate that constructing models only with Multi-Layer Perceptrons (MLPs) and skip connections, alleviating the utilization of self-attention layers, might yield favorable outcomes in vision tasks (Tolstikhin et al., 2021).

Despite encouraging outcomes in visual recognition evaluations and the advantages of a global receptive field, many proposed MLP token or channel mixer architectures were hand-engineered for specific purposes. These can be primarily categorized into four categories (Liu R. et al., 2022): spatial projection, axial projection, channel projection, and shifting-based. Table 1 provides a taxonomy of these mixer families along with their key characteristics and representative models. Spatial projection mixers [e.g., MLP-Mixer (Tolstikhin et al., 2021), ResMLP (Touvron et al., 2022), gMLP (Liu H. et al., 2021)] apply dense MLPs across all token positions to achieve a global receptive field, but incur *O*(*N*^2^) complexity in the number of tokens and are resolution-dependent. Axial projection mixers [e.g., Vision Permutator (Hou et al., 2022)] apply projections along individual spatial axes independently, reducing complexity but limiting cross-axis interaction. Channel projection mixers focus mixing operations on the channel dimension only, without capturing spatial relationships. Shifting-based mixers [e.g., CycleMLP (Chen et al., 2021), HireMLP (Guo et al., 2022), AS-MLP (Lian et al., 2021), S^2^-MLP (Yu et al., 2022)] rearrange or shift elements of the feature tensor, making them inherently resolution-insensitive and computationally efficient relative to the other types.

**Table 1:** Taxonomy of MLP-mixer families by mixing type.

Mixing type	Representative models	Complexity	Res.-Indep.	Receptive field
Spatial projection	MLP-Mixer (Tolstikhin et al., 2021), ResMLP (Touvron et al., 2022), gMLP (Liu H. et al., 2021)	*O*(*N*^2^)	No	Global
Axial projection	Vision permutator (Hou et al., 2022)	$O\left(N\sqrt{N}\right)$	Partial	Axial
Channel projection	Channel-only MLPs	*O*(*NC*^2^)	Yes	None (spatial)
Shifting-based	CycleMLP (Chen et al., 2021), HireMLP (Guo et al., 2022), AS-MLP (Lian et al., 2021), S^2^-MLP (Yu et al., 2022)	*O*(*NC*^2^)	Yes	Local-to-global

“Res.-Indep.” indicates whether the architecture is resolution-independent (can handle variable input sizes). Complexity refers to the spatial mixing operation.

Notably, Basis-Driven Learnable Operator (BDLO) is not proposed as a new standalone architecture that competes with state-of-the-art CNNs [e.g., ConvNeXt (Liu Z. et al., 2022)] or Vision Transformers [e.g., Swin Transformer (Liu Z. et al., 2021), DeiT (Touvron et al., 2021)]. Instead, BDLO is a *drop-in replacement operator* for the shifting block in existing shifting-based MLP-mixer architectures. Our comparisons are therefore between the original shifting operations and the BDLO-approximated operations within the same architecture, rather than between BDLO and fundamentally different model families. We focus on the shifting-based family because these architectures are already resolution-insensitive and have lower baseline complexity, making them the most natural candidates for further simplification via learned basis decomposition. Nevertheless, we note that CycleMLP has been shown to achieve competitive performance with Swin Transformer on dense prediction benchmarks (Chen et al., 2021). AS-MLP obtains results comparable to transformer-based architectures at similar FLOPs (Lian et al., 2021), confirming that the shifting-based family represents a relevant and competitive class of models.

This study builds on the shifting type of MLP-mixers, which is inherently resolution-insensitive (Liu R. et al., 2022), and they have operations that can be approximated through different mathematical operations as detailed later in the paper.

Other studies approached the problem from different perspectives, aiming for a more general dynamic approach. The DynaMixer estimates a mixing matrix from the input across the patch and channel dimensions (Wang et al., 2022). The Active Token Mixer estimates an index mixing vector and mixes patches accordingly (Wei et al., 2023). The Vision-Permutator combines three mixing paths, including an identity (Hou et al., 2022). All these studies focused on adding more capacity to the model to learn different components of the mixing process, such as the mixing matrix, or on adding all possible combinations for mixing.

Based on the aforementioned discussion, we identify a research gap: approximated, learnable mixing with less capacity. Hence, our proposed BDLO for MLP-mixers goes in a different direction than dynamic networks. BDLO aims to learn coefficients for a linear combination of real, discrete, complete bases to reduce the number of parameters in shifting-based mixers. Several fixed bases' spectral transformations, such as Discrete Cosine Transform (DCT), Discrete Fourier Transform (DFT), Wavelets, and Butterfly approximations, were considered in several representation problems (Parker, 1995; Dao et al., 2021). Also, statistical spectral feature extraction methods such as Principal Component Analysis (PCA) or Linear Discriminant Analysis (LDA) provide transformations related to the data to provide better transformations in a given sense (Bishop and Nasrabadi, 2006). Later on, sparse representation learning using either Dictionary Learning (Xu et al., 2017) for synthesis or Transform Learning (Ravishankar and Bresler, 2012) for analysis was investigated in the literature. For mixing, we consider the bases for representing a general spectral transformation matrix, which are essentially the standard basis matrices. We also compare the performance with DCT bases, since they are real and complete, which turns out to be almost identical to that of the general bases.

This study attempts to reach a balance between specialized solutions, which achieve better results due to the introduction of knowledge into the architecture design and add fewer requirements on the models' capacities, and the flexibility of adding dense layers with full global receptive fields to learn from the data. The proposed solution is to learn approximations for the shifting-based mixing operations using a general spectral transformation, and allow the model to learn the proper decomposition of the input into those bases for mixing.

The main contributions of this paper can be summarized as follows: (1) Proposing a novel BDLO for MLP-mixers for image recognition, (2) Achieving similar performance with fewer parameters and FLOPs, and (3) Providing the theoretical derivation for the new proposed operator.

The remainder of the paper is organized as follows: Section 2 introduces the proposed spectral transformation operator. Section 3 discusses the experimental results on CIFAR10, CIFAR100, and reduced ImageNet of size 32 and with 500 classes following (Chrabaszcz et al., 2017). Finally, the conclusion is given in Section 4.

## 2 The proposed basis-driven learnable operator

Although the main advantage of the MLP-mixer architecture is its global receptive field, it makes it difficult for the network to learn on its own the different sizes and scales of image details using dense MLPs. Hence, the main shortcoming of MLPs is the lack of communication across different image patches to enable abstraction of feature representations. This is why the original paper (Tolstikhin et al., 2021) suggested the mixing part. Several studies that came afterward suggested different mixing approaches that enable intercommunication across tokens (image patches) and channels.

To clarify the overall framework, a standard MLP-mixer block processes its input through four sequential stages: (i) a patch embedding that projects non-overlapping image regions into a token-channel table *X* ∈ ℝ^*h*×*w*^, (ii) a token-mixing (spatial mixing) operation that shuffles or shifts information across spatial positions, (iii) a channel-mixing MLP applied independently at each position, and (iv) skip connections with layer normalization. In shifting-based mixers (Liu R. et al., 2022), stage (ii) is realized by predetermined rearrangement of elements in *X*. Our proposed BDLO targets *only* this stage, learning a data-dependent transformation matrix *P* = *D*·LinearFlatten(σ(*WX*)) that replaces the fixed shifting pattern. Because only *d* scalar coefficients need to be learned equal to the number of bases, this integration directly reduces the parameter count and computational cost of the mixing layer (1.68 GFLOPs vs. 3.33–5.08 GFLOPs for the original layers), while the remaining stages (i), (iii), and (iv) are preserved unchanged and continue to provide the representational capacity of the original architecture.

The literature varies in its mixing perspectives, from fully automated free-parameter search to restricted hand-engineered mixing operations. Each approach has its pros and cons, which balance between enabling a wider yet more complex search space vs. a more restricted yet simpler one. Several MLP-mixer architectures have proposed well-engineered solutions for the mixing component, which involves shuffling the different elements of image tensors in a predetermined way, and have achieved respectable results (Liu R. et al., 2022).

Existing shifting operations involve swapping or shuffling of different elements in both the rows and columns in a specific way. We build our BDLO approximation based on the following arguments: any element interchanging operation can be approximated by a permutation matrix, and any sequence of permutation matrices is also a permutation matrix (Strang, 2007). Applying the permutations to both rows and columns means doing multiplication from both sides of the matrix under consideration (Dummit and Foote, 2004).

The BDLO seeks to balance both perspectives by using a semi-restricted search space: learning a continuous transformation matrix that approximates the shifting permutation by learning only the coefficients of a generic real discrete complete basis to form a transformation matrix for mixing. Permutation matrices are a strict subset of real transformation matrices: a permutation is the special case in which *n* single-entry standard bases are combined with unit coefficients, one per row and column. BDLO instead assigns continuous coefficients σ(*WX*) ∈ (0, 1) over the complete basis, so the learned *P* is a continuous transformation in the span of the basis rather than a strict permutation. The intermediate case, a generalized (monomial) matrix with a single non-zero but non-unit entry per row and column, is the product of a permutation and a diagonal matrix and therefore performs a weighted permutation (reordering with scaling); this clarifies how relaxing the unit-value constraint still preserves the reordering action. No strict-permutation, or doubly-stochastic, constraint is imposed; the relaxation is deliberate for differentiability and input adaptivity. A detailed proof of this claim is available in the Appendix.

The BDLO primarily replaces the shifting block inside existing MLP-mixer architectures such as CycleMLP (Chen et al., 2021), HireMLP (Guo et al., 2022), and AS-MLP (Lian et al., 2021) while keeping all other components such as patch embedding, channel-mixing MLPs, skip connections, and classification head unchanged.

### 2.1 The spectral transformation MLP layer

**Figure 1:**
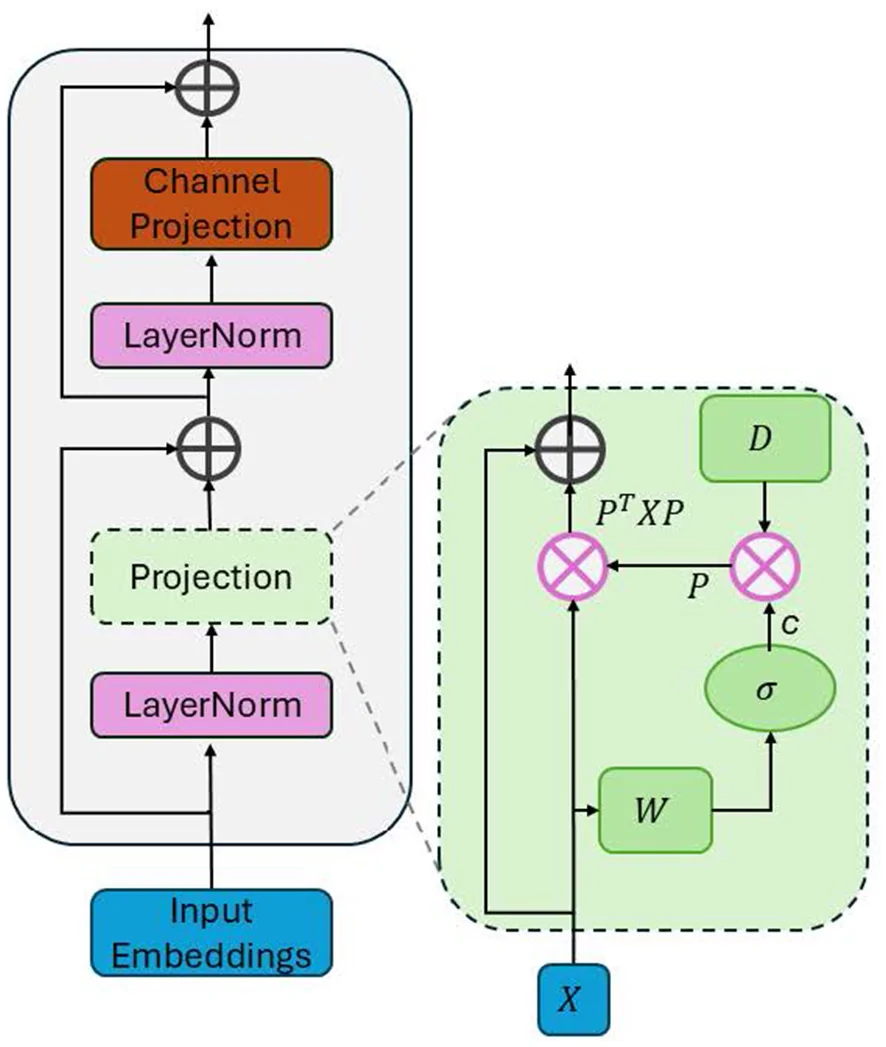
Diagrammatic representation of the BDLO.

Figure 1 shows a graphical representation of the proposed BDLO. The variables used are those detailed below in this section, with the symbol ⊗ indicating multiplication and ⊕ indicating addition. Following the same block design as the previously proposed mixer architectures under study, we only change the shifting block for mixing. As with any general form of row and column operations using a permutation matrix, *P*^*T*^*XP* operates on both rows and columns for an input matrix *X* ∈ ℝ^*h*×*w*^, where *h* and *w* are the input dimensions. In the general (non-square) case, the row and column reorderings are realized by two distinct matrices, $X^{\prime }=P_{h}^{T}XP_{w}$ with $P_{h}\in {\mathbb{R}}^{h\times h}$ permuting rows and $P_{w}\in {\mathbb{R}}^{w\times w}$ permuting columns, exactly as derived in the Appendix ($A^{\prime }=P_{\pi }AQ_{\kappa }$). When *h* = *w*, as for the square token grids produced by the patch embedding in the studied mixers, the two collapse to a single matrix *P* ∈ ℝ^*h*×*h*^ and the operator simplifies to the symmetric form *P*^*T*^*XP* used here; *P* is then a transformation matrix with the same dimensions as the (square) input. However, the proposed BDLO estimates *P* from the input itself by P=D·LinearFlatten(σ(WX)), where *D* ∈ ℝ^*h*×*w*×*d*^ is the fixed-bases tensor, *W* ∈ ℝ^*w*×*h*^ is a learnable weight matrix, and *LinearFlatten*() converts a matrix to a vector with optional reduction of dimensions to *d* from *h*×*w*. In our experiments, we use *d* = 256. Without loss of generality, we assume the standard basis set is used, where each basis has a single non-zero entry. This can be replaced by any fixed-basis real discrete transformation that spans ℝ^*h*×*w*^ for mixing. We test this empirically using the DCT bases. We emphasize the distinction between completeness and the coefficient reduction: the basis tensor *D* is *complete*, since the standard (or DCT) bases span ℝ^*h*×*w*^ and can represent any target transformation exactly, whereas projecting the coefficient vector to *d* = 256≪*h*×*w* through a deliberate *low-rank approximation* of that complete expansion rather than a full expansion. This reduction is supported empirically by the low sensitivity to *d* (Section 3.6) and theoretically by the low effective spectral rank of the sparse permutation targets (Section 3.8). Because *D* and *W* are instantiated at the token-table dimensions of each stage, and shifting-based mixers are themselves resolution-insensitive, the operator adapts to a different input size simply by instantiating *D* and *W* at the corresponding (*h, w*); it is therefore not restricted to square feature maps or to a fixed input size.

The derivation of the derivative assuming sigmoid non-linearity σ is as follows:


$$\begin{array}{l} \;X^{\prime \prime }=X^{\prime }+X=P^{T}XP+X\\ \frac{\delta X^{\prime \prime }}{\delta W}=\frac{\delta P^{T}XP}{\delta W}=\frac{\delta P^{T}XP}{\delta c}*\frac{\delta c}{\delta W}\\ \;=\left(\frac{\delta P^{T}}{\delta c}XP+P^{T}X\frac{P}{\delta c}\right)*\frac{\delta c}{\delta W} \end{array}$$ (1)

By substituting *P* = *Dc*, the derivative reduces to Equation 2.


$$\begin{array}{l} \frac{\delta X^{\prime \prime }}{\delta W}=\left(D^{T}XDc+c^{T}D^{T}XD\right)*\sigma \left(WX\right)\left(I-\sigma \left(WX\right)\right)*X^{T} \end{array}$$ (2)

### 2.2 Baseline shifting-based MLP-mixers

**Figure 2:**
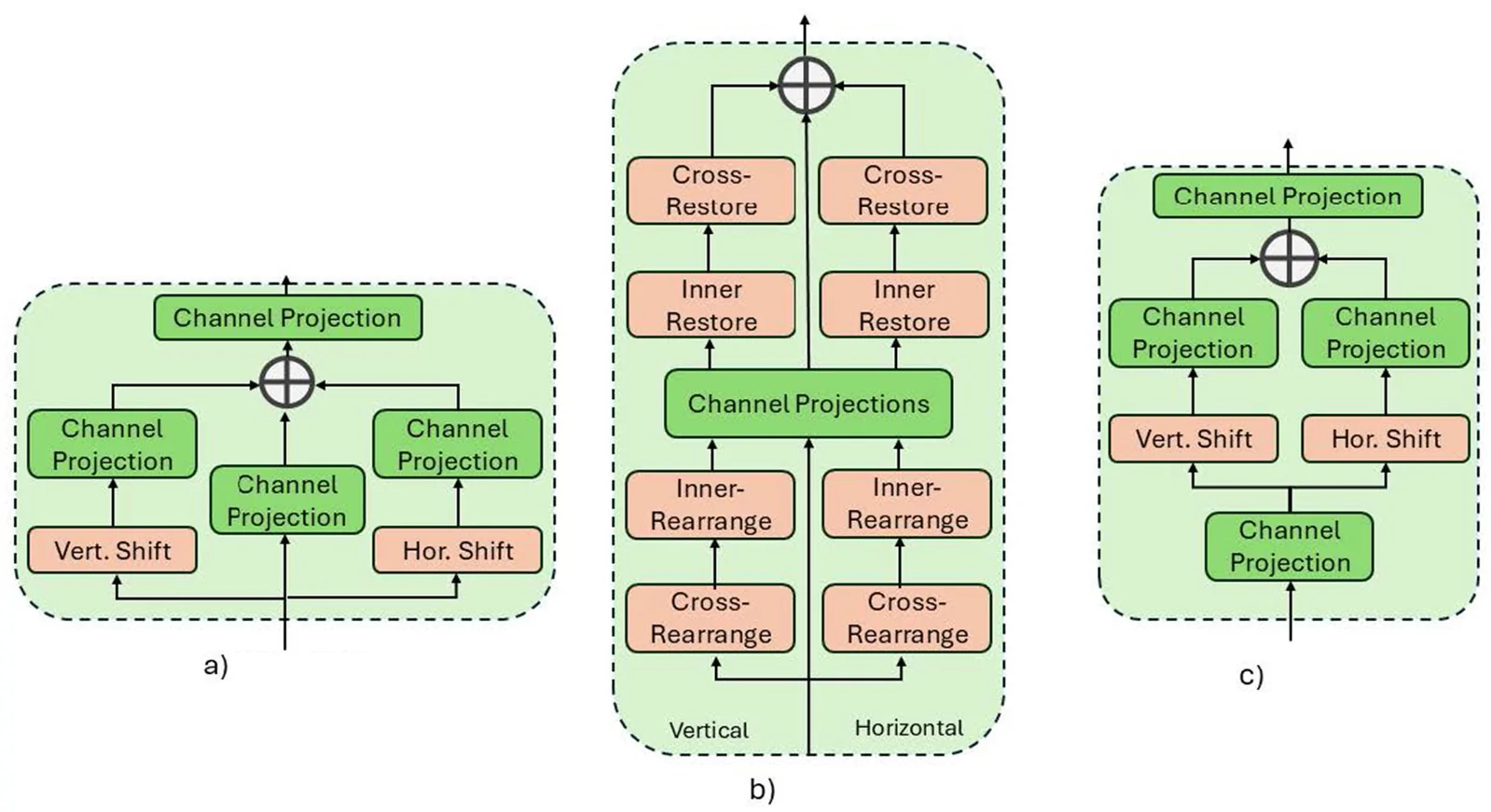
High-level computational graphs for shifting-based MLP-mixers, with orange operations indicating shuffling and shifting operations. **(a)** CycleMLP. **(b)** HireMLP. **(c)** AS-MLP.

In this subsection, we describe the three shifting-based MLP-mixer architectures used as baselines for evaluating the proposed BDLO. All three belong to the shifting category of MLP-mixers (Liu R. et al., 2022), which achieves spatial mixing by rearranging or shifting elements of the feature tensor rather than applying dense projections or self-attention. This makes them inherently resolution-insensitive and less complex than spatial or channel projection alternatives. We select these architectures because BDLO is designed as a drop-in replacement for their shifting blocks; all other components (patch embedding, channel-mixing MLPs, skip connections, and classification head) remain unchanged. Figure 2 shows the high-level computational graphs for each baseline model. In the following subsections, we assume *X* ∈ ℝ^*h*×*w*×*C*^ denote the input feature map, where *h*, *w*, and *C* are the height, width, and channel dimensions, respectively, and let $W_{\text{fc}}\in {\mathbb{R}}^{C\times C}$ denote a generic fully-connected (channel-mixing) weight matrix.

#### 2.2.1 CycleMLP

CycleMLP (Chen et al., 2021) introduces the Cycle Fully-Connected (Cycle FC) layer for spatial mixing. The core idea is to aggregate features from spatially offset positions via cyclic shifting with learnable step sizes, applied in parallel branches. Let *K* denote the number of parallel branches (typically *K* = 3). For the *k*-th branch, a step-size pair $\left(s_{h}^{\left(k\right)},s_{w}^{\left(k\right)}\right)$ defines the spatial offset. The Cycle FC operation for branch *k* at spatial position (*i, j*) and output channel *c*′ is:


$$\begin{array}{l} Y_{i,j,c^{\prime }}^{\left(k\right)}=\overset{C}{\underset{c=1}{\sum }}X_{\left(i+s_{h}^{\left(k\right)}\cdot c\right)\;\mathrm{mod}\;h,\left(j+s_{w}^{\left(k\right)}\cdot c\right)\;\mathrm{mod}\;w,c}\cdot W_{\text{fc}}^{\left(k\right)}\left(c^{\prime },c\right) \end{array}$$ (3)

where the modular arithmetic enforces cyclicity, making the operation resolution-insensitive with a linear computational complexity of *O*(*hwC*^2^) per branch. The typical configuration uses step sizes (1, 0), (0, 1), and (0, 0) corresponding to vertical, horizontal, and identity branches, with larger step sizes such as (7, 0) and (0, 7) available for wider receptive fields. The outputs of the *K* branches are summed with a residual connection, as given in Equation 4:


$$\begin{array}{l} X^{\prime }=\overset{K}{\underset{k=1}{\sum }}Y^{\left(k\right)}+X \end{array}$$ (4)

This multi-branch design, inspired by the factorization of convolutions, captures both local and semi-global spatial dependencies. CycleMLP builds a family of models (B1–B5) in PVT-style and has demonstrated competitive performance with Swin Transformer on dense prediction tasks while maintaining fewer FLOPs. In our experiments, we use the B1, B2, and B3 variants.

In our formulation (Section 2), the cyclic shifting in each branch can be viewed as a specific permutation matrix *P*^(*k*)^ acting on the spatial indices, where ${\left(P^{\left(k\right)}\right)}_{ij}=1$ if position *j* maps to position *i* under the cyclic offset $\left(s_{h}^{\left(k\right)},s_{w}^{\left(k\right)}\right)$. The BDLO replaces the *K* fixed permutation matrices with a single learned transformation *P* = *D*·*c* that approximates the combined effect.

#### 2.2.2 HireMLP

HireMLP (Guo et al., 2022) proposes a Hierarchical Rearrangement (Hire) module for spatial mixing. Its mixing block comprises three independent branches: two spatial branches (one along height, one along width) and one channel-mixing branch. Each spatial branch performs a two-level rearrangement as follows.

##### Inner-region rearrangement

Taking the height direction as an example, the input *X* ∈ ℝ^*h*×*w*×*C*^ is partitioned into *g* non-overlapping spatial groups along the height dimension, yielding *X* = [*X*_1_, *X*_2_, …, *X*_*g*_] where each $X_{l}\in {\mathbb{R}}^{h_{r}\times w\times C}$ with region size *h*_*r*_ = *h*/*g*. Inside each region *X*_*l*_, the tokens are rearranged (shuffled) along the height axis by a fixed inner-region permutation $P_{\text{in}}\in {\mathbb{R}}^{h_{r}\times h_{r}}$:


$$\begin{array}{l} {\tilde{X}}_{l}=P_{\text{in}}X_{l},\;l=1,\ldots ,g \end{array}$$ (5)

This enables local information exchange within each spatial region.

##### Cross-region rearrangement

To enable global communication, a circular shift of magnitude δ is applied along the height direction across all regions, corresponding to a global permutation $P_{\text{cross}}\in {\mathbb{R}}^{h\times h}$:


$$\begin{array}{l} {\hat{X}}_{i,j,c}={\tilde{X}}_{\left(i+\delta \right)\;\mathrm{mod}\;h,j,c} \end{array}$$ (6)

The rearranged features are then processed by two channel-mixing fully-connected layers with a non-linearity, as given in Equation 7:


$$\begin{array}{l} Z_{\text{height}}=W_{\text{fc},2}\sigma_{g}\left(W_{\text{fc},1}\hat{X}\right) \end{array}$$ (7)

where σ_*g*_ denotes the GELU activation (Hendrycks and Gimpel, 2016). The same two-level procedure is applied independently along the width direction to produce *Z*_width_, with its own inner-region and cross-region permutations.

##### Channel branch and aggregation

A third branch applies a direct channel-mixing FC layer *Z*_ch_ = *W*_ch_*X* without any spatial rearrangement. The three branches are aggregated with a residual connection:


$$\begin{array}{l} X^{\prime }=Z_{\text{height}}+Z_{\text{width}}+Z_{\text{ch}}+X \end{array}$$ (8)

Notably, the channel branch *Z*_ch_ introduces cross-channel communication that is absent in purely spatial shifting operations. This is directly relevant to our results, since the BDLO—operating only on the spatial dimensions via the row-column permutation *P*^*T*^*XP*—cannot approximate this channel-mixing component, which explains the larger accuracy gap observed for HireMLP (Section 3.7). In our experiments, we use the Tiny, Small, and Base variants.

#### 2.2.3 AS-MLP

The Axial Shifted MLP (AS-MLP) (Lian et al., 2021) achieves spatial mixing by shifting feature channels along the height and width axes independently. Given the input *X* ∈ ℝ^*h*×*w*×*C*^ and a shift size *s*, the channels are partitioned into 2*s*+1 groups of size *C*/(2*s*+1) each. For the horizontal (width) shift, the *m*-th group is shifted by an offset (*m*−*s*−1) positions:


$$\begin{array}{l} {\hat{X}}_{i,j,c}^{\left(m\right)}=X_{i,\left(j+m-s-1\right)\;\mathrm{mod}\;w,c},\;c\in G_{m},\;m=1,\ldots ,2s+1 \end{array}$$ (9)

where $G_{m}$ is the index set of channels in the *m*-th group. The same procedure is applied along the height (vertical) direction independently. The shifted features from both directions are concatenated and processed by a channel-projection MLP, as given in Equation 10:


$$\begin{array}{l} X^{\prime }=W_{\text{fc},2}\sigma_{g}\left(W_{\text{fc},1}\left[{\hat{X}}_{\text{h}}||{\hat{X}}_{\text{w}}\right]\right)+X \end{array}$$ (10)

where ${\hat{X}}_{\text{h}}$ and ${\hat{X}}_{\text{w}}$ denote the horizontally and vertically shifted features, || denotes concatenation along the channel dimension, and σ_*g*_ is the GELU activation (Hendrycks and Gimpel, 2016). The computational complexity is *O*(*hwC*^2^), driven by the four channel projections (two per direction), without any multiplication or addition cost for the shift itself.

In the BDLO framework, each axial shift can be represented as a sparse permutation matrix: the horizontal shift corresponds to $P_{\text{w}}\in {\mathbb{R}}^{w\times w}$ applied to rows, and the vertical shift to $P_{\text{h}}\in {\mathbb{R}}^{h\times h}$ applied to columns, with both applied independently per channel group. The BDLO collapses this group-wise, axis-decomposed structure into a single learned transformation *P* = *D*·*c* applied uniformly as *P*^*T*^*XP*. Due to AS-MLP's high model capacity, it tends to overfit on smaller datasets such as CIFAR100, which has only 600 training samples per class (Tan, 2024). This is an important consideration when interpreting comparative results (Section 3.3). In our experiments, we use the Tiny, Small, and Base variants.

### 2.3 Baseline shifting operation comparison summary

Table 2 provides a unified comparison of the projection mechanisms employed by each baseline and the proposed BDLO, summarizing the key differences in how spatial mixing is achieved.

**Table 2:** Comparison of projection mechanisms across baseline architectures and the proposed BDLO.

Method	Spatial Op.	Channel mix in shift	GFLOPs	Parameterization
CycleMLP (Chen et al., 2021)	*K* parallel cyclic shifts	No	5.08	Fixed step sizes $\left[s_{H}^{\left(k\right)},s_{W}^{\left(k\right)}\right]$
HireMLP (Guo et al., 2022)	2-level hierarchical rearrange	Yes (*Z*_ch_ branch)	3.52	Fixed inner/cross permutations + FC
AS-MLP (Lian et al., 2021)	Group-wise axial shifts	No	3.33	Fixed per-group offsets along *H*, *W*
**BDLO (ours)**	Learned *P*^*T*^*XP*	No	**1.68**	*d* coefficients via *D*·σ(*WX*)

The table highlights two distinguishing properties of BDLO: (i) it is the only method whose mixing pattern is *adaptive* to the input (the transformation matrix *P* is computed from *X* via the learnable weight *W* and sigmoid activation), and (ii) it achieves the lowest computational cost by replacing multi-branch or multi-group operations with a single basis-coefficient multiplication. The trade-off is that BDLO does not include channel-wise mixing within the shifting block (unlike HireMLP's *Z*_ch_ branch), which explains the performance gap observed for HireMLP in Section 3.7.

We further clarify the precise sense in which BDLO corresponds to the differently named baseline shifts. BDLO replaces a single per-channel spatial shift and operates only on the spatial dimensions through *P*^*T*^*XP*; it supports no inter-channel shift. Despite their different names, the spatial operations of all three baselines are the same elementary primitive, a per-channel reordering of spatial indices (a composition of row/column permutations): the multi-branch cyclic shift of CycleMLP (Equation 3), the group-wise axial shift of AS-MLP (Equation 9, whose group-wise offsets vary the shift amount by channel without moving information between channels), and the inner- and cross-region rearrangements of HireMLP (Equations 5, 6). By the Appendix result that any sequence of row and column permutations collapses to a single left/right pair, these are all parameterizations of one primitive that the single learned *P*^*T*^*XP* approximates. The only baseline component outside this primitive is HireMLP's channel-mixing branch *Z*_ch_ (Equation 8), which mixes across channels and therefore cannot be reproduced by the spatial-only operator, explaining its larger gap.

## 3 Experimental results

In this section, we evaluate the proposed BDLO as a drop-in replacement for the shifting blocks in CycleMLP (Chen et al., 2021), HireMLP (Guo et al., 2022), and AS-MLP (Lian et al., 2021). All experiments are trained from scratch on a single NVIDIA RTX 4090 24GB GPU. We organize the evaluation into computational complexity (Section 3.1), training setup (Section 3.2), performance on CIFAR10/100 (Section 3.3), performance on ImageNet (Section 3.4), basis choice analysis (Section 3.5), hyperparameter sensitivity (Section 3.6), and failure cases and robustness (Section 3.7).

### 3.1 Computational complexity comparison

We first compare the computational cost of the proposed BDLO layer against the original mixing layers. Table 3 reports the number of floating-point operations (GFLOPs) computed using a simulated input of size 1 × 128 × 224 × 224 (batch, embedding channels, height, and width). The BDLO layer achieves 1.68 GFLOPs, representing a 2–3 × reduction compared to the baseline layers. This reduction is expected because BDLO replaces the hand-engineered shifting operations with a compact set of learned coefficients over a fixed basis, avoiding the multi-branch parallel processing (CycleMLP), hierarchical rearrangement (HireMLP), or sequential axial shifts (AS-MLP) used in the original designs.

**Table 3:** Computational complexity comparison for the different mixing layers vs. BDLO, measured in GigaFLOPs (GFLOPs) using a simulated input of size 1 × 128 × 224 × 224.

Layer	GFLOPs
CycleMLP (Chen et al., 2021)	5.08 (3 × )
HireMLP (Guo et al., 2022)	3.52 (2.1 × )
AxialShift (Lian et al., 2021)	3.33 (2 × )
BDLO (proposed)	1.68

The increase factor relative to BDLO is shown in parentheses.

The per-layer figures above are incurred once per block, not just once overall: the three baselines are four-stage pyramids that stack many blocks, and each block contains exactly one spatial-mixing (shift) module together with one channel-mixing MLP. The shift module that BDLO replaces therefore recurs once per block, 10–37 times depending on the variant, as summarized in Table 4 (depths taken from the original papers Chen et al., 2021; Guo et al., 2022; Lian et al., 2021). Consequently, the 2–3 × per-layer reduction accumulates with depth, which is what produces the substantial whole-model parameter reductions reported in Table 5 (from −21.8% for CycleMLP-B1 up to −56.7% for HireMLP-Small), while the remaining components (patch embeddings, channel MLPs, and classification head) are shared between each original architecture and its BDLO variant. Comparing each module against the per-block channel-mixing MLP, whose cost at the same 1 × 128 × 224 × 224 configuration is ≈13.2 GFLOPs for the standard expansion-ratio-4 design. The shifting module therefore accounts for approximately 28% (CycleMLP, 5.08 GFLOPs), 21% (HireMLP, 3.52), and 20% (AS-MLP, 3.33) of the per-block compute, which BDLO (1.68) reduces to ≈11%. Since this module recurs at every block (Table 4), the reduction applies throughout the network.

**Table 4:** Per-stage block counts for the studied baselines (depths from the original papers). Each block contains one spatial-mixing (shift) module, so the shift operator that BDLO replaces recurs once per block.

Architecture	Variant	Stage depths [*L*_1_, *L*_2_, *L*_3_, *L*_4_]	Shift modules
CycleMLP (Chen et al., 2021)	B1/B2/B3	[2, 2, 4, 2]/[2, 3, 10, 3]/[3, 4, 18, 3]	10/18/28
HireMLP (Guo et al., 2022)	Tiny/Small/Base	[2, 2, 4, 2]/[3, 4, 10, 3]/[4, 6, 24, 3]	10/20/37
AS-MLP (Lian et al., 2021)	Tiny/Small/Base	[2, 2, 6, 2]/[2, 2, 18, 2]/[2, 2, 18, 2]	12/24/24

**Table 5:** Top-1 test accuracy (%) comparison between the proposed BDLO and the original implementations on CIFAR10 and CIFAR100 (Krizhevsky, 2009), with relative change shown in parentheses.

Model	Size	Params (M)	CIFAR100	TR CIFAR100	CIFAR10	TR CIFAR10
CycleMLP	B1	14.7	67.0	4.59	90.1	2.75
	B1-BDLO	11.5 (–21.8%)	66.3 (–1%)		89.5 (–0.6%)	
	B2	26.3	67.5	7.76	90.9	9.05
	B2-BDLO	20.2 (–23.2%)	66.3 (–1.8%)		89.0 (–2.1%)	
	B3	37.9	66.5	3.88	91.3	4.74
	B3-BDLO	29.1 (–23.2%)	65.9 (–0.9%)		90.3 (–1.1%)	
HireMLP	Tiny	17.5	63.2	16.50	90.4	5.00
	Tiny-BDLO	10.5 (–40%)	59.0 (–6.6%)		88.6 (–2%)	
	Small	32.6	63.6	4.23	89.8	4.76
	Small-BDLO	14.1 (–56.7%)	62.1 (–2.4%)		87.4 (–2.7%)	
	Base	57.7	63.5	8.37	90.3	7.27
	Base-BDLO	31.5 (–45.4%)	61.1 (–3.8%)		87.3 (–3.3%)	
AS-MLP	Tiny	27.5	63.7	1.47	85.7	-1.47
	Tiny-BDLO	20.0 (–27.3%)	63.4 (–0.4%)		86.1 (+0.4%)	
	Small	48.8	62.7	–19.64	85.7	0.00
	Small-BDLO	35.4 (–27.5%)	66.1 (+5.4%)		85.7 (0%)	
	Base	86.7	56.0	–67.94	85.1	–0.70
	Base-BDLO	61.8 (–28.7%)	66.9 (+19.5%)		85.3 (+0.2%)	

“Params (M)” denotes millions of parameters. TR is the Trade-off Ratio: (relative accuracy loss/relative parameter reduction) × 100; lower is better, and negative values indicate BDLO gains accuracy.

### 3.2 Training configuration

Table 6 details the hyperparameter settings used across all CIFAR and reduced ImageNet experiments. The configuration follows modern training practices including cosine learning rate scheduling with warm-up (Loshchilov and Hutter, 2016), label smoothing (Müller et al., 2019), AutoAugment (Cubuk et al., 2018), and gradient clipping. These settings are applied identically to both the original architectures and their BDLO counterparts, ensuring a fair comparison. For the full ImageNet1K experiment, the same configuration is used with a batch size of 256.

**Table 6:** Training configuration for all experiments. The same settings are applied to both original and BDLO variants to ensure a fair comparison.

Hyperparameter	Value/range
Learning rate	2.00E-03
Weight decay	0.05
Epochs	300
Learning rate scheduler	Cosine scheduler (Loshchilov and Hutter, 2016)
Warm up epochs	10
Cool down epochs	10
Min learning rate	1.00E-05
Loss function	Cross-entropy
Label smoothing (Müller et al., 2019)	0.1
Augmentation	AutoAugment (Cubuk et al., 2018)
Gradient clip	1
Validation split	0.1
Multiplicative noise probability	0.5

### 3.3 Performance on CIFAR10/100

Table 5 compares top-1 test accuracy (%) and parameter counts (in millions) for the original architectures and their BDLO counterparts on CIFAR100 and CIFAR10 (Krizhevsky, 2009). The different model sizes (B1–B3 for CycleMLP; Tiny, Small, Base for HireMLP, and AS-MLP) are those defined in the original publications. Models with the suffix “BDLO” use our proposed operator in place of the original shifting block, with relative changes in parameters and accuracy shown in parentheses.

We also report the Trade-off Ratio (TR), defined as the ratio of relative accuracy loss to the relative reduction in the number of parameters, multiplied by 100. A lower TR indicates a more favorable efficiency-accuracy trade-off; a negative TR indicates that BDLO achieves both fewer parameters *and* higher accuracy than the original.

Across all configurations, the mean difference in accuracy between BDLO and the originals is 0.38% in favor of BDLO, while the mean reduction in parameters is 12.84M. The maximum TR values are 16.5 (CIFAR100, HireMLP-Tiny) and 9.05 (CIFAR10, CycleMLP-B2).

To assess statistical significance, we apply the Wilcoxon signed-rank test at the 5% significance level. The *p*-value for the number of parameters is 0.0039, confirming that BDLO produces models from a significantly different (lower) parameter distribution. The *p*-values for test accuracy are 0.496 (CIFAR100) and 0.0625 (CIFAR10), both failing to reject the null hypothesis, and indicating no statistically significant difference in performance between the original and BDLO variants.

### 3.4 Performance on ImageNet

#### 3.4.1 Reduced ImageNet (32 × 32, 500 classes)

Table 7 reports the top-1 test accuracy (%) for the Tiny variants on a reduced version of ImageNet1K, downsampled to 32 × 32 resolution with 500 classes following the protocol of Chrabaszcz et al. (2017) using Lanczos downsampling. We adopt the same training configuration as in Table 6, with a batch size of 256. This reduced benchmark is used because training all 18 model variants (3 architectures × 3 sizes × 2 variants) from scratch on full-resolution ImageNet1K would require prohibitive compute on our single-GPU setup. The protocol of Chrabaszcz et al. (2017) has been adopted in prior works for resource-constrained benchmarking.

**Table 7:** Top-1 test accuracy (%) for the Tiny variants of the original models vs. their BDLO counterparts on the reduced ImageNet (32 × 32, 500 classes) (Chrabaszcz et al., 2017).

Model	Type	Top-1 Accuracy (%)
CycleMLP (Chen et al., 2021)	Original	51
	BDLO	52 (+1.96%)
HireMLP (Guo et al., 2022)	Original	45
	BDLO	42 (−6.67%)
AS-MLP (Lian et al., 2021)	Original	49
	BDLO	43 (–12.2%)

Relative change is indicated in parentheses.

The results are consistent with the CIFAR findings in Table 5. The Wilcoxon test yields a *p*-value of 0.5, indicating no statistically significant performance difference. However, a notable gap appears for AS-MLP: its high-capacity benefits from the larger sample count in ImageNet (approximately 2,500 images per class for 500 classes) compared to CIFAR100 (600 per class). With more training data, the original AS-MLP can exploit its full capacity, whereas BDLO's lower-capacity approximation is less able to close the gap. This contrasts with the CIFAR100 results where AS-MLP overfits, and BDLO actually outperforms it (Tan, 2024). Importantly, this reduced benchmark already provides cross-architecture coverage at ImageNet scale: all three baseline families (CycleMLP, HireMLP, AS-MLP) and their BDLO variants are evaluated here (Table 7), so the ImageNet-scale comparison is not limited to a single model.

#### 3.4.2 Full ImageNet1K

To validate that findings from the reduced dataset generalize to the standard benchmark, we also evaluate AS-MLP Tiny on the full ImageNet1K (Deng et al., 2009) using the same training configuration as above. The original model achieves 60.2% top-1 and 82.2% top-5 accuracy, while the BDLO variant achieves 60.6% top-1 and 82.4% top-5 accuracy. This result confirms that, at the Tiny scale, BDLO can match or slightly exceed the original even on full-resolution, full-class ImageNet, while using 27.3% fewer parameters. We present this full-resolution result as a single-configuration transfer-validation: because all three families are evaluated on the reduced benchmark, and that benchmark is shown here (via AS-MLP-Tiny) to be predictive of full-scale behavior, the reduced-ImageNet results also fortify the claims for CycleMLP and HireMLP. Evaluating additional full-resolution configurations was not feasible on our single RTX 4090, and we flag this single full-scale configuration as a limitation to be broadened in future study.

### 3.5 Basis choice analysis

The theoretical framework (Section 2 and Appendix) establishes that any real complete discrete basis can represent the target permutation matrix via a change-of-basis similarity transform. To verify this empirically, we repeated all experiments using DCT bases (Ahmed et al., 1974) instead of the default standard (single-entry) bases. The results are nearly identical: the average cosine distance between the learned coefficient vectors of the two basis types is 0.0049, and both coefficient distributions are approximately normal. This confirms that the modeling capacity of BDLO is invariant to the choice of basis, as predicted by the theory, and that the learning process successfully finds an equivalent decomposition regardless of the basis representation.

### 3.6 Hyperparameter sensitivity analysis

**Figure 3:**
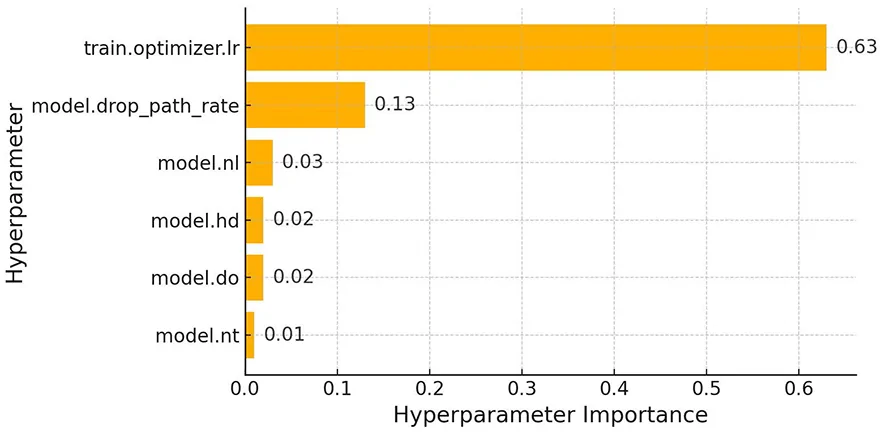
Hyperparameter sensitivity analysis using Optuna (Akiba et al., 2019) for CIFAR100 on CycleMLP with BDLO. lr, learning rate; do, dropout rate; nl, number of layers in the BDLO estimation network; nt, multiplication order in Equation 1, hd, hidden dimension of the BDLO estimation network.

To identify which hyperparameters most influence BDLO's performance, we conduct a sensitivity analysis using Optuna (Akiba et al., 2019) with the Tree-structured Parzen Estimator (TPE) (Watanabe, 2023) on CycleMLP with BDLO for CIFAR100 (Krizhevsky, 2009). Table 8 lists the Optuna configuration, and Table 9 defines the search space. Figure 3 shows the results.

**Table 8:** Optuna configuration for the hyperparameter sensitivity study.

Parameter	Value
Seed	12345
Consider prior	TRUE
Prior weight	1
Magic clip	TRUE
Endpoints	FALSE
Expected improvement candidates	10
Trials	50

**Table 9:** Hyperparameter search space for the sensitivity analysis.

Hyperparameter	Search space
Hidden dimension (hd)	{32, 64, 128, 256, 512, 1024}
Number of layers (nl)	[2, 16]
Multiplication order (nt)	{1, 2}
Dropout (do)	{0, 0.1, 0.25, 0.5}
Drop path rate	[0, 0.8]
Learning rate (lr)	[2e-4, 2e-2]

“Hidden Dimension” and “Number of Layers” refer to the BDLO estimation network; “Multiplication Order” corresponds to the exponent in the transformation (*P*^*T*^*XP* for order 2, *PX* for order 1).

The analysis reveals that the model is primarily sensitive to the learning rate and the path drop rate, while BDLO-specific parameters (hidden dimension, number of layers, and multiplication order) have comparatively low impact. This is theoretically justified: the shifting operations correspond to sparse permutation matrices, which are binary linear transformations. By the universal approximation theorem (Cybenko, 1989), a single hidden layer with arbitrary width suffices to approximate such binary functions, explaining the low sensitivity to the number of layers and hidden dimensions of the BDLO estimation network.

### 3.7 Failure cases and robustness analysis

The most prominent failure case is HireMLP, where BDLO exhibits the largest accuracy drops (up to −6.6% for HireMLP-Tiny on CIFAR100, TR = 16.5). This is attributable to HireMLP's use of a dedicated channel-mixing branch alongside its spatial rearrangement branches (Guo et al., 2022). Since BDLO operates exclusively on the spatial dimensions (approximating row and column permutations via *P*^*T*^*XP*), it cannot capture the cross-channel communication that HireMLP's third branch provides. This represents a fundamental scope limitation of the current BDLO formulation rather than a failure of the approximation itself.

The experiments span three distinct data regimes: CIFAR10 (6,000 samples per class, 10 classes), CIFAR100 (600 samples per class, 100 classes), and reduced ImageNet (approximately 2,500 samples per class, 500 classes). BDLO's relative performance is consistent across these regimes for CycleMLP and HireMLP. For AS-MLP, an interesting data-dependent pattern emerges. On the smaller CIFAR100, the original AS-MLP overfits due to its high capacity (Tan, 2024), and BDLO actually outperforms it substantially (e.g., +19.5% for AS-MLP Base). On the larger ImageNet, the original can better exploit its capacity, and the gap favors the original (−12.2% for AS-MLP Tiny). This is consistent with BDLO providing an implicit regularization effect through its constrained parameterization, which is beneficial in low-data regimes but may limit expressiveness when abundant data is available.

**Figure 4:**
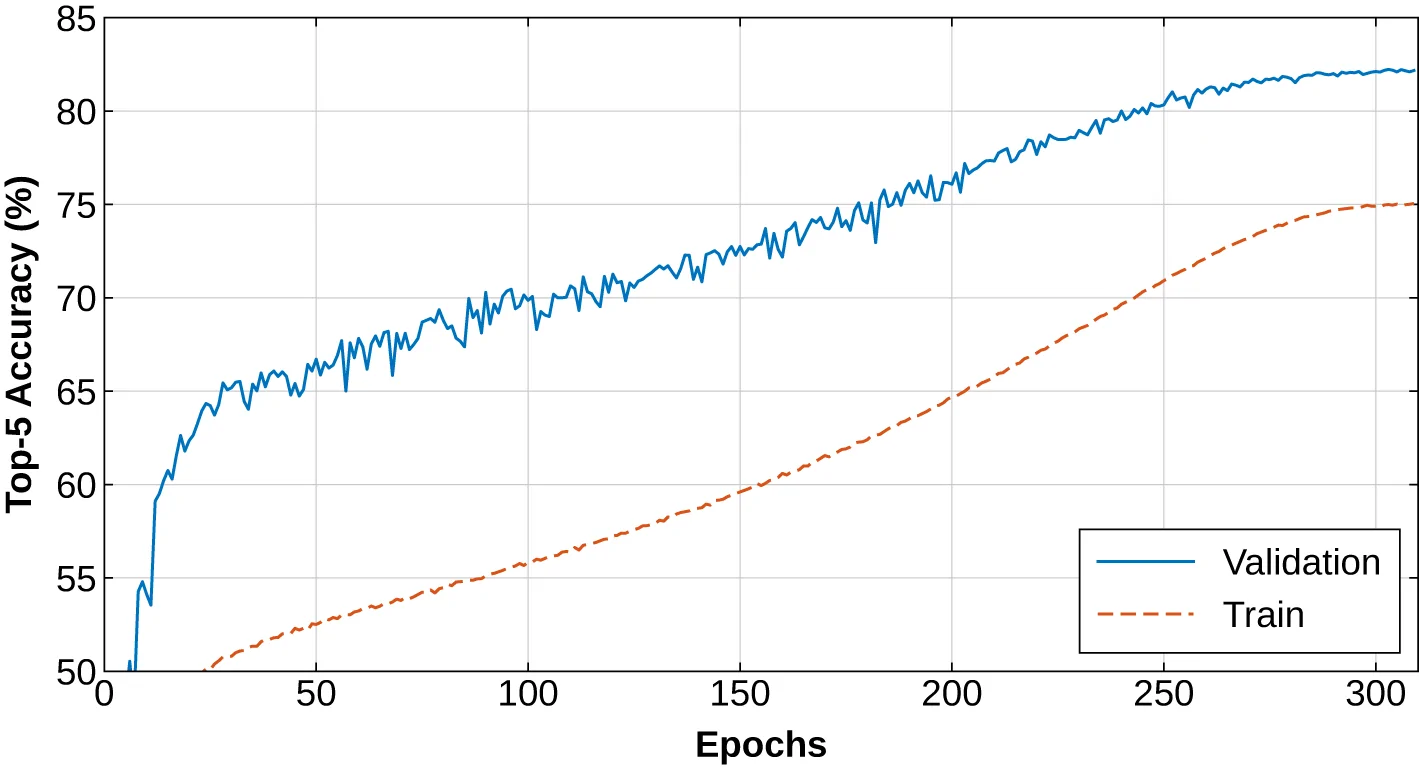
Training and validation top-5 accuracy of the original AS-MLP-Tiny on ImageNet1K. The validation accuracy stays above the training accuracy throughout, an expected consequence of train-time regularization and augmentation that is disabled at evaluation (Müller et al., 2019; Cubuk et al., 2018; Kornblith et al., 2021); the faster late-epoch improvement of the training curve relative to the validation curve indicates overfitting onset, consistent with AS-MLP's high capacity (Tan, 2024).

Direct training dynamics support this reading. The training and validation curves for the original AS-MLP-Tiny on ImageNet1K (Figure 4, Appendix) show the validation top-5 accuracy lying above the training top-5 accuracy throughout (e.g., 0.652 vs. 0.517 near epoch 38 and about 0.82 vs. 0.75 at convergence, the former matching the 82.2% reported above). A validation-above-training ordering is expected here rather than anomalous: the regularizers in our configuration (Table 6), namely label smoothing (Müller et al., 2019), AutoAugment (Cubuk et al., 2018), and drop-path, are active only during training and are known to lower the measured training accuracy relative to evaluation, an effect documented on ImageNet (Kornblith et al., 2021). Overfitting is nonetheless visible in the improvement rates: in the later epochs the training curve climbs faster than the validation curve (which flattens), so the high-capacity model fits the training data increasingly faster than it generalizes, consistent with AS-MLP's tendency to overfit (Tan, 2024).

All 18 CIFAR models and both ImageNet configurations converged without training divergence across 300 epochs. The BDLO operator introduces no additional instability: the sigmoid activation bounds the learned coefficients to [0, 1], the skip connection (*P*^*T*^*XP*+*X*) ensures gradient flow even when the learned transformation is suboptimal, and gradient clipping (set to 1) prevents exploding gradients. No additional stabilization techniques were required beyond the standard training recipe detailed in Table 6.

### 3.8 Interpretation of BDLO

The BDLO constructs its transformation matrix as a weighted superposition $P=\sum_{k=1}^{d}c_{k}D_{k}$, where the coefficients *c*_*k*_ = [LinearFlatten(σ(*WX*))]_*k*_ are derived from the input itself. The sigmoid activation constrains each *c*_*k*_ to [0, 1], which bounds the spectral norm of *P* and ensures that the coefficient vector varies smoothly with the input. This smoothness provides implicit regularization: abrupt changes in the mixing pattern between similar inputs are discouraged, and noisy coefficient values are suppressed by the sigmoid's saturation. This mechanism helps explain why BDLO outperforms the original AS-MLP on CIFAR100 (up to +19.5% for AS-MLP Base, Table 5), where the high-capacity original overfits on only 600 samples per class (Tan, 2024), while the sigmoid-bounded coefficients prevent the learned transformation from fitting training noise. The approximately normal distribution of the learned coefficients (Section 3.5) further confirms that the model converges to smooth, well-behaved solutions rather than sparse or degenerate ones. This interpretation also connects to the gated spectral modulation framework of Wang et al. (2026), who showed that architectures where the gating signal derives from the input itself consistently reinforce salient variations rather than suppressing them, which is achieved by the BDLO's self-gating structure σ(*WX*).

When the DCT basis (Ahmed et al., 1974) is used, this interpretation becomes precisely spectral. Each coefficient *c*_*k*_ controls the amplitude of a specific spatial-frequency mode in *P*, so the BDLO performs learned frequency-domain filtering where low-frequency coefficients govern coarse spatial rearrangements and high-frequency coefficients control fine-grained local permutations. The near-identical results between standard and DCT bases (cosine distance 0.0049) confirm that both parameterizations traverse the same space of achievable transformations, related by the change-of-basis similarity $SP_{\rho }S^{-1}$ (Appendix). This basis invariance, together with the low sensitivity to *d* observed in the hyperparameter sensitivity analysis in Section 3.6, indicates that the target shifting operations have low effective spectral rank, which is consistent with their nature as sparse permutation matrices, so even a compact coefficient vector captures the dominant mixing modes. Taken together, these observations characterize the BDLO as an *input-adaptive spectral modulator*: the fixed basis *D* defines the coordinate system, while the data-dependent coefficients σ(*WX*) act as a learned filter that selectively activates different mixing modes according to the input content, achieving the essential spatial communication of hand-engineered shifts with fewer parameters and built-in regularization.

## 4 Conclusion

This study is another attempt to exploit the potential of MLP-Mixers to enable deep learning applications in image recognition. Our results show that some attempts to develop hand-engineered mixing operations published in the literature can be approximated by a BDLO for MLP-Mixers of tiny to medium sizes on medium-sized datasets. The reduction in the number of required parameters is, on average, 12.84M parameters on the CIFAR100 dataset, while retaining an average performance difference of 0.38% in favor of the proposed BDLO approximation. We note that this comparability is an average behavior in the parameter-constrained regime: HireMLP, whose mixing includes a dedicated channel-mixing branch, and AS-MLP on the larger ImageNet show model-specific accuracy drops, so BDLO is best positioned as an efficient replacement for shifting-based mixers at small-to-medium scale rather than a universally superior operator. This study is the start of a line of research on network simplification to enable dense architectures such as MLP-mixers to be more implementation-friendly, requiring fewer customized operations and less tuning for the different applications.

### 4.1 Future research

Although our proposed BDLO is shown to be a good alternative for shifting-type mixer operations, it remains limited to that type of mixing operation. Future research can generalize the learning operators to other types of mixing operations. This will be challenging because there is no direct mapping to the non-linear mixing operations used, but the approximation can be a learnable transformation that uses a pre-trained library of mixing operations for a new problem. Other studies have worked on approximating transformations and weight matrices in NNs, such as Parker (1995); Dao et al. (2021). Another promising direction is to investigate the properties of the learned weight matrices in MLP-mixers and study the benefits of compression or approximation using orthogonal or over-complete bases, for example, for sparsity. This could also extend to challenging applications such as medical applications, aerial unmanned vehicles, and compression-resistant watermarking for learning models (Nie et al., 2024; Trigka and Dritsas, 2025). Moreover, integrating BDLO into detection and segmentation pipelines is a natural next step. Evaluating BDLO-equipped backbones on COCO and ADE20K would directly measure whether the classification-level findings transfer to dense prediction.

## Data Availability

The original contributions presented in the study are included in the article/supplementary material, further inquiries can be directed to the corresponding author.

## Appendix: Permutation matrices with different bases approximation

In this section, we show that any finite sequence of row and column reorderings of a matrix can be realized as left- and right-multiplications by permutation matrices, and that these permutation matrices can be computed from the standard basis and from any real ordered basis of the ambient space. A permutation matrix is a square *n*×*n* matrix with exactly one entry of 1 in each row and each column and zeros elsewhere, and satisfies *P*^−1^ = *P*^*T*^, so permutation matrices are orthogonal.

For *n* ∈ ℕ and a permutation ρ ∈ *S*_*n*_, define the permutation matrix $P_{\rho }\in {\mathbb{R}}^{n\times n}$ by

$${\left(P_{\rho }\right)}_{ij}=\{\begin{array}{ll} 1, & j=\rho (i),\\ 0, & \text{otherwise}, \end{array}\text{ equivalently }P_{\rho }e_{i}=e_{\rho (i)}.$$

Let *A* ∈ ℝ^*m*×*n*^. If *P* ∈ ℝ^*m*×*m*^ is a permutation matrix for some π ∈ *S*_*m*_, then left-multiplication permutes rows:

$${\left(PA\right)}_{ij}=\overset{m}{\underset{k=1}{\sum }}P_{ik}A_{kj}=A_{\pi^{-1}\left(i\right),j},$$

so the rows of *A* are reordered by π. If *Q* ∈ ℝ^*n*×*n*^ is a permutation matrix for some κ ∈ *S*_*n*_, then right-multiplication permutes columns:

$${\left(AQ\right)}_{ij}=\overset{n}{\underset{k=1}{\sum }}A_{ik}Q_{kj}=A_{i,\kappa^{-1}\left(j\right)},$$

so the columns of *A* are reordered by κ.

Consider any finite sequence of row permutations (π_1_, …, π_*r*_)⊂*S*_*m*_ and column permutations (κ_1_, …, κ_*s*_)⊂*S*_*n*_ with associated permutation matrices *P*_π_*k*__ and *Q*_κ_ℓ__. Applying them in order yields

$$A^{\prime }=\left(P_{\pi_{r}}\cdots P_{\pi_{1}}\right)A\left(Q_{\kappa_{1}}\cdots Q_{\kappa_{s}}\right)=P_{\text{row}}AQ_{\text{col}},$$

where *P*_row_ = *P*_π_*r*__⋯*P*_π_1__ = *P*_π_ for the composed permutation π = π_*r*_°⋯°π_1_, and *Q*_col_ = *Q*_κ_1__⋯*Q*_κ_*s*__ = *Q*_κ_ for κ = κ_1_°⋯°κ_*s*_. Since permutation matrices form a group under multiplication and satisfy *P*^−1^ = *P*^*T*^, the composition law shows any finite sequence of row/column reordering collapses to left/right multiplication by a single pair of permutation matrices.

To compute these matrices from the standard basis, obtain *P*_ρ_ and *Q*_κ_ by permuting rows (or columns) of the identity matrix: the *i*-th row of *P*_ρ_ has its 1 in column ρ(*i*), equivalently *P*_ρ_*e*_*i*_ = *e*_ρ(*i*)_. For a matrix *A* ∈ ℝ^*m*×*n*^, use $P_{\pi }\in {\mathbb{R}}^{m\times m}$ on the left to permute rows and $Q_{\kappa }\in {\mathbb{R}}^{n\times n}$ on the right to permute columns, so any combined reordering equals $A^{\prime }=P_{\pi }AQ_{\kappa }$.

For computation from an arbitrary real ordered basis *B* = (*v*_1_, …, *v*_*n*_) of ℝ^*n*^, define the linear operator *T*_*B*_ by *T*_*B*_(*v*_*i*_) = *v*_ρ(*i*)_ for a chosen ρ ∈ *S*_*n*_. The matrix of *T*_*B*_ with respect to the basis *B* is exactly the permutation matrix *P*_ρ_ (same 0–1 pattern in that basis). If *S* = [*v*_1_⋯*v*_*n*_] is the change-of-basis matrix giving coordinates of *B* in the standard basis, then the matrix of the same operator in standard coordinates is

$${\left[T_{B}\right]}_{\text{std}}=S{\left[T_{B}\right]}_{B}S^{-1}=SP_{\rho }S^{-1},$$

which realizes the same permutation of the basis vectors in ambient coordinates via the similarity transform induced by the change of basis.
